# Recent Advances in Solid Catalysts Obtained by Metalloporphyrins Immobilization on Layered Anionic Exchangers: A Short Review and Some New Catalytic Results

**DOI:** 10.3390/molecules21030291

**Published:** 2016-02-29

**Authors:** Shirley Nakagaki, Karen Mary Mantovani, Guilherme Sippel Machado, Kelly Aparecida Dias de Freitas Castro, Fernando Wypych

**Affiliations:** 1 Laboratório de Bioinorgânica e Catálise, Departamento de Química, Centro Politécnico, Universidade Federal do Paraná (UFPR), Curitiba, Paraná 81531-990, Brazil; karen.mary16@gmail.com (K.M.M.); guismachado@yahoo.com.br (G.S.M.); kedc2000@yahoo.com.br (K.A.D.F.C.); wypych@ufpr.br (F.W.); 2 Centro de Estudos do Mar, Pontal do Paraná, Paraná, Universidade Federal do Paraná (UFPR), Paraná 83255-000, Brazil; 3 Departamento de Química e QOPNA, Universidade de Aveiro, Aveiro 3810-193, Portugal

**Keywords:** porphyrin, oxidation, layered double hydroxides, layered hydroxide salts, immobilization, heterogeneous catalysis

## Abstract

Layered materials are a very interesting class of compounds obtained by stacking of two-dimensional layers along the basal axis. A remarkable property of these materials is their capacity to interact with a variety of chemical species, irrespective of their charge (neutral, cationic or anionic). These species can be grafted onto the surface of the layered materials or intercalated between the layers, to expand or contract the interlayer distance. Metalloporphyrins, which are typically soluble oxidation catalysts, are examples of molecules that can interact with layered materials. This work presents a short review of the studies involving metalloporphyrin immobilization on two different anionic exchangers, Layered Double Hydroxides (LDHs) and Layered Hydroxide Salts (LHSs), published over the past year. After immobilization of anionic porphyrins, the resulting solids behave as reusable catalysts for heterogeneous oxidation processes. Although a large number of publications involving metalloporphyrin immobilization on LDHs exist, only a few papers have dealt with LHSs as supports, so metalloporphyrins immobilized on LHSs represent a new and promising research field. This work also describes new results on an anionic manganese porphyrin (MnP) immobilized on Mg/Al-LDH solids with different nominal Mg/Al molar ratios (2:1, 3:1 and 4:1) and intercalated with different anions (CO_3_^2−^ or NO_3_^−^). The influence of the support composition on the MnP immobilization rates and the catalytic performance of the resulting solid in cyclooctene oxidation reactions will be reported.

## 1. Introduction

### 1.1. Metalloporphyrins and Homogeneous Catalysis

Porphyrins are highly conjugated heterocyclic macrocycles derived from four pyrrole rings interconnected by four methine bridges [1]. The chemical and physical properties of these compounds allow for their use in various fields [1,2]. In fact, porphyrins have promising medicinal [3] and catalytic [4] applications.

Porphyrin macrocycles can coordinate different catalytically active metals, like iron, copper, and nickel, among others. The catalytic use of the resulting metal complex will depend on the selected metal. Metalloporphyrins (MPs) occur in the nuclei of several oxidation enzymes such as cytochromes P450 [5,6], peroxidases [6], and catalases; they are often employed as model catalysts [7].

The great versatility of reactions catalyzed by P450 biomimetic systems; e.g., alkane hydroxylation and olefin epoxidation, regio- and stereo-selective reactions, and drug metabolism, has motivated researchers to develop new porphyrin derivatives [8,9].

Groves [10] performed the pioneered studies on MPs as catalysts and showed that the Fe^3+^ complex [Fe(TPP)Cl] can mimic cytochrome P450 in many reactions, mainly hydroxylation and epoxidation, which boosted research in this field [4,5,8].

MPs can efficiently and selectively catalyze a series of oxidation reactions in homogeneous medium (*i.e.*, when the catalyst, substrate, and products are in the same phase). However, depending on the porphyrin structure, secondary reactions (MP destructive oxidation by other previously activated MP and secondary MP–MP interaction reaction; e.g., MP dimerization by μ-oxo bridges) may take place in homogeneous medium, to deactivate the catalytic species [11,12]. Moreover, homogeneous systems preclude catalyst recovery and reuse [13,14].

To overcome these common issues in homogeneous medium, various research groups have attempted to design catalysts for heterogeneous processes by immobilizing MPs on inorganic or organic solid supports [11,15,16,17,18]. MP immobilization should facilitate catalyst recovery and reuse as well as minimize destructive oxidation and other deactivating processes, ultimately preventing catalyst efficiency loss.

A variety of solid matrixes can serve as supports for MPs; for example, silicas, zeolites, polymeric nanoparticles, dendrimers, natural and synthetic layered hydroxides, and fibrous compounds, among others.

### 1.2. Metalloporphyrins and Heterogeneous Catalysis

In 1983, van der Made and co-workers [15] published one of the first reports on MP immobilization with a view to stabilizing the MP complex and promoting catalyst recycling; the catalyst support consisted of a polymeric matrix. These researchers realized that isolating the MP active site by immobilizing a MnP on a polymer support (isocyanide) was possible and avoided formation of the less reactive dimers observed during homogeneous catalysis. In fact, the immobilized MnP was three times more active for cyclohexene epoxidation than the free MnP [15].

Since then, a large number of inorganic solids have been studied as supports for MPs and many other complexes for catalytic purposes. Examples of these solids include clay minerals [19,20,21], silica [22,23], and metal oxides [24,25], among others [26]. Besides, polymeric matrixes like isocyanide [15], poly(vinyl alcohol) [27], and polystyrene [28] have also been employed to immobilize MPs and obtain efficient catalysts for oxidation reactions.

MP immobilization can also avoid the undesired approach between catalytic species and the consequent catalyst destruction. Moreover, proper combination of MP and support mimics the protein cavity of the natural enzyme and modulates specific selectivity for various catalytic reactions [19,29].

Facile catalyst recovery from the heterogeneous catalytic reaction solution is among the advantages of catalyst immobilization and enables catalyst recycling. Catalyst recovery often demands just simple filtration or centrifugation [11,19].

In this context, layered double hydroxides (LDHs) and layered hydroxide salts (LHSs) have emerged as interesting supports to immobilize MPs. These matrixes are easy to obtain by one-pot synthesis, and their variable chemical composition is based on environmentally friendly elements. Additionally, they have layered morphology, controllable particle size, inertia in different experimental conditions, and low cost.

Layered compounds can interact with MPs not only at the crystal basal surface, but also at the crystal edge. MPs can even intercalate between the matrix layers. All these alternatives expand the applicability of this class of compounds. When MPs intercalate between LDH or LHS layers [30,31,32], the resulting solid displays distinct and unusual selectivity as compared to the catalyst in solution (homogeneous catalysis) or even to the MP immobilized on the layered compound surface [33,34].

This short review offers a brief overview of the articles about MP immobilization on two different layered compounds, LDHs and LHSs, published over the past years. It also includes new data for an MP immobilized on different Mg/Al-LDHs.

### 1.3. Layered Compounds

Layered compounds are part of an unusual class of compounds obtained by packing of two-dimensional units (layers) along the basal crystallographic axis. These compounds have called researchers’ attention over the last years mainly because it is possible manipulate single layers, but this manipulation is not possible on the traditional tridimensional materials.

Depending on the layer charges, layered compounds of synthetic or natural origin can be classified into neutral, positive, or negative. These charges will depend on the material that is present during the genesis or the oxi-reduction process in which the metal cation undergoes oxidation or reduction, to intercalate cations or anions, respectively [35]. In some examples, neutral species can also intercalate between the layers by just breaking the bonds that hold the layers together (e.g., kaolinite intercalated with different neutral molecules).

The layer thickness will depend on the material used to prepare the layered compound, and the material may be one-, three-, or seven-atom-thick as in the case of graphite, transition metal dichalcogenides, and 2:1 cationic exchange clay minerals or micas, respectively.

Anionic Exchange layered compounds are rare in nature, but it is possible to obtain countless compounds of this type in the laboratory by using simple synthetic procedures. Most of these compounds are based on the Brucite structure (Mg(OH)_2_), which is common for calcium, manganese, iron, cobalt, and nickel hydroxides, all of which bear a double positive charge.

**Figure 1:**
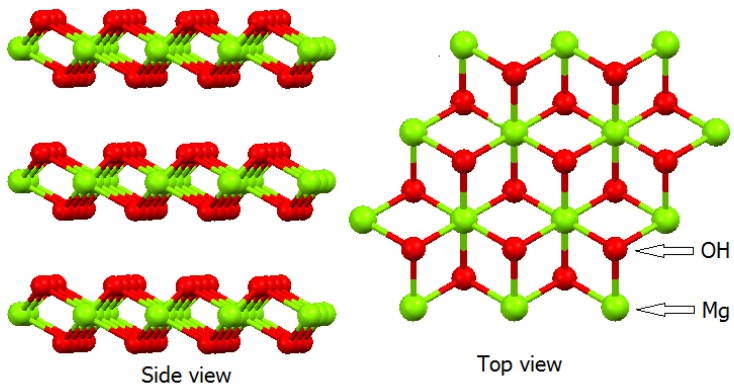
Brucite structure. Hydrogen atoms were removed from the structure to facilitate visualization [36,37].

The Brucite structure consists of layers of slightly distorted octahedra. In the center of these octahedral Mg^2+^, cations are coordinated with six hydroxyl groups occupying the vertices of the octahedra. In turn, each hydroxyl group coordinates with three Mg^2+^ cations, to generate a tri-octahedral structure where all the octahedral sites of the structure are filled. The octahedral unit shares vertices with three adjacent octahedra connected by the corners (Figure 1) the 2+ charge of the Mg^2+^ cation is shared by six bonds with the hydroxyls, to give a charge of (1/3)+. Because three Mg^2+^ bonds share the single charge on hydroxyl, the result is (1/3)−. Therefore, the (1/3)+ and (1/3)− charges will cancel each other out [36,37].

Another common example of simple hydroxide is Gibbsite (Al(OH)_3_). Because Gibbsite contains Al^3+^ in its structure, one third of the octahedral positions remain unoccupied, to ensure structural neutrality. This arrangement gives rise to the structure called di-octahedral. Charge balance for these structures is based on the Pauling electrostatic valence theory, which postulates that each cation present in the polyhedra tends to compensate the electric charge of each anion in stable coordinated structures. These layers bind to each other by Van der Waals forces. This interaction furnishes three-dimensional crystals that tend to grow preferentially along the layer planes, to afford crystals with plaque morphology.

The majority of layered compounds occurring in the mineral form have neutral structure or constitute cationic exchangers; e.g., clay minerals belonging to the 2:1 group. However, layered minerals with anionic exchange properties are rare. Examples of such minerals are hydrotalcite-like compounds, also known as layered double hydroxides (LDHs).

The LDH structure resembles the structure of the mineral Brucite, in which trivalent cations can isomorphically substitute the Mg^2+^ cations, to generate a positively charged residue. The presence of anions in the LDH interlayer space compensates for this charge and renders these compounds their characteristic anionic exchange capacity.

The formula [M^2+^_1−x_M^3+^_x_(OH)_2_]^x+^·A^m−^_x/m_·nH_2_O represents the LDH composition, where M^2+^ refers to a divalent metallic cation (e.g., Mg^2+^, Zn^2+^, *etc*.), M^3+^ corresponds to a trivalent metallic cation (e.g., Al^3+^, Fe^3+^, *etc*.), and A^m−^ represents an intercalated hydrated anion with charge m^−^ (e.g., NO_3_^−^, CO_3_^2−^, *etc*.). Although LDH are rare in nature, synthesizing these compounds in the laboratory is easy and relatively inexpensive [38,39,40].

To be part of the LDH structure, the metallic cations M^2+^ and M^3+^ must remain in the center of a hydroxyl-coordinated octahedron, which implies in a limited range of ionic radii. In general, the molar ratio between the di- and trivalent metallic cations in the LDH (M^2+^/M^3+^) can vary from 1 to 8, which corresponds to 0.5 > x > 0.11 in the general LDH formula. Because each trivalent cation accounts for the excess positive charge in the layers, the x value determines the LDH charge density.

Higher charge density in the layer increases the amount of intercalated anions for each divalent cation, an equivalent amount of anions should intercalate between the LDH layers. Hence, increased M^2+^/M^3+^ molar ratio lowers the charge density and the number of intercalated anions. Consequently, the anions become sufficiently separated, which minimizes interaction between the pillared layers and facilitates delamination and exfoliation. Intercalated anions can be organic or polymeric, for example, which aids tailoring of the LDH properties to the desired application. When a low proportion of organic species intercalates between the layers, neutral organic species can occupy the spaces available between the organic anions, to cause a phenomenon called adsolubilization.

**Figure 2:**
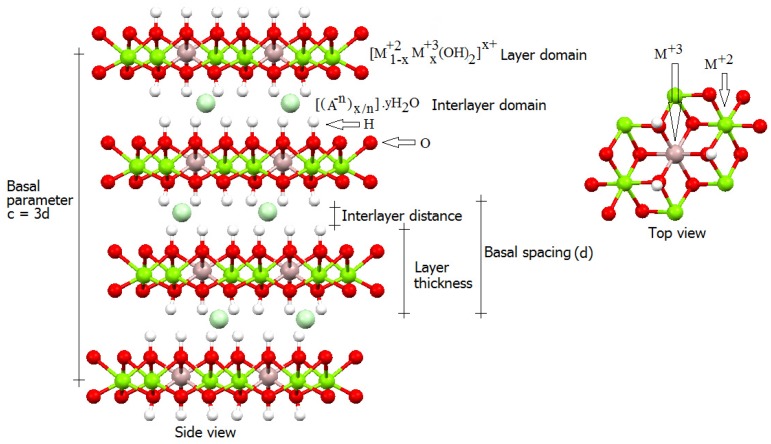
Schematic representation of a generic 3R Layered Double Hydroxide (LDH) structure [36,37].

Figure 2 shows a schematic representation of an LDH structure of the 3R polytype (three layers along the basal axis) according to the nomenclature of Ramsdell.

Some literature reports have mentioned that M^4+^ cations can also substitute M^2+^ metals in LDHs, but the literature lacks unequivocal confirmation of these structures.

**Figure 3:**
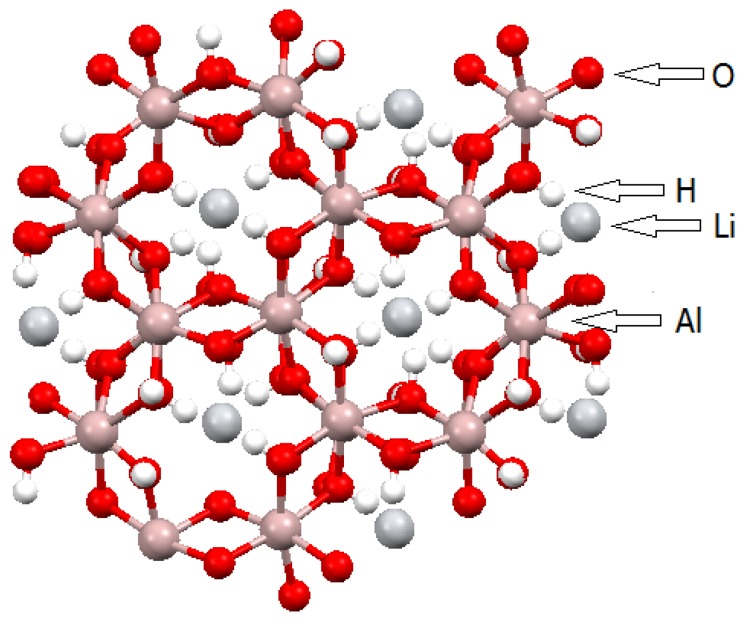
Top view of a single [LiAl_2_(OH)_6_]A·zH_2_O layer (A = Cl^−^, Br^−^, NO_3_^−^, *etc*.) [36,37].

A distinct example of LDH is the structure of Gibbsite (Al(OH)_3_). This compound reacts with excess lithium salts to form compounds similar to LDH of the type Li_0.5_Al(OH)_3_(A)_0.5_·yH_2_O or [LiAl_2_(OH)_6_]A·zH_2_O (A = Cl^−^, Br^−^, NO_3_^−^, *etc*.) [41]. These compounds are impossible to be synthesized by the regular precipitation route. Indeed, only the reaction of Gibbsite with lithium salts, either in solution or after mechanochemical reaction, yields these compounds. The lithium cations most likely occupy an octahedral site of the Gibbsite structure (Gibbsite is a di-octahedral structure where one octahedral site of the layers is empty), and the hydrated anion intercalates in the interlayer space (Figure 3). Besides Gibbsite, the same type of reaction can also occur for a mixture of Nordstrandite and Bayerite, which bear similar structures [42].

Specific literature review papers exist for the majority of these layered compounds, but a class of layered materials with anionic exchange capacity, denominated layered hydroxide salts (LHSs) and which are rare as mineral, will also be the focus of the present review article.

LHSs are compounds that resemble LDHs, but another mechanism rather than M^2+^ substitution with M^3+^ generates the excess positive charge in its layers. The general formulation of LHSs is M^2+^(OH)_2−x_(A^n−^)_x/n_·yH_2_O, where M^2+^ is a metallic cation (e.g., Ni^2+^ Zn^2+^, Ca^2+^, Cd^2+^, Co^2+^, or Cu^2+^), and A is a counter anion with charge n^−^. (Ex.: NO_3_^−^, SO_4_^2−^, CH_3_COO^−^, *etc.* [43,44]).

**Figure 4:**
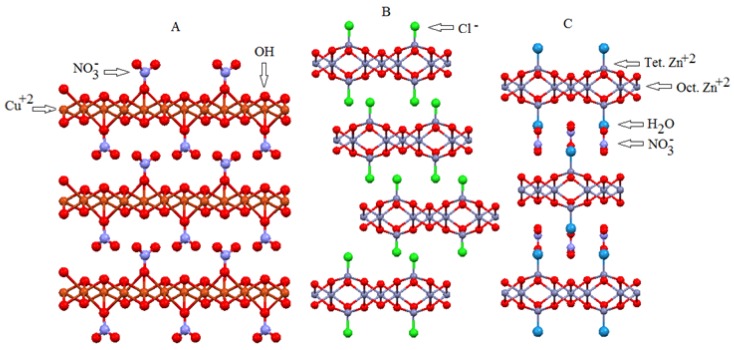
Side view of the structures of: (**A**) Copper hydroxite nitrate; (**B**) Zinc hydroxide chloride; (**C**) Zinc hydroxide nitrate. Hydrogen atoms were removed from the structure to facilitate visualization [36,37].

The cationic layers in the LHS and the LDH structures have distinct generation mechanism. In LHSs, other anions occupy a fraction of the hydroxide sites, as in the case of Cu_2_(OH)_3_NO_3_. Two different polymorphs exist in this phase. One polymorph is orthorhombic, as reported for the structure of Gerhardtite and other synthetic monoclinic compounds. In the orthorhombic structure of Cu_2_(OH)_3_NO_3_, nitrate anions substitute one fourth of the hydroxide anions and coordinate with three different Cu^2+^ cations, to occupy octahedral sites (Figure 4A). Although nitrate anions are grafted to the layers, they can be replaced by means of anion exchange reactions. In the case of the second polymorph, one fourth of the Zn^2+^ ions present in the octahedral sites are removed from the structure, and two tetrahedral sites are generated in the upper and lower octahedral empty site. The anions are grafted directly to the tetrahedral Zn^+2^ sites, as observed for the mineral Simonkolleite (Zn_5_(OH)_8_Cl_2_·2H_2_O) (Figure 4B). A third polymorph in which a structure similar to Simonkolleite contains water molecules directly bound to the tetrahedral Zn^2+^ sites is also possible, but it requires the presence of a counter anion in the second coordination sphere, to neutralize the positive charges of the layers (e.g., Zn_5_(OH)_8_(NO_3_)_2_·2H_2_O) (Figure 4C).

The structure of the aforementioned zinc hydroxide nitrate Zn_5_(OH)_8_(NO_3_)_2_·2H_2_O can be considered a structural variation of the Zn(OH)_2_ structure, where one fourth of the octahedral Zn^2+^ cations are removed from the layer. Every octahedral site occupied by a Zn^2+^ cation shares edges with two empty and four occupied octahedra, producing a layer with a residual negative charge ([Zn_3_(OH)_8_]^2−^). To compensate for the deficit in positive charge, tetrahedral Zn^2+^ cations accommodate below and above the empty octahedra of the layer. In this way, three corners of the tetrahedra coordinate with oxygen atoms of the layer, whereas the fourth position coordinates with a water molecule. In this configuration, the layer is positively charged [Zn_3(oct.)_(OH)_8_Zn_2(tet.)_(H_2_O)_2_]^2+^, where “oct.” and “tet.” represent the Zn^2+^ cations located in octahedral and tetrahedral sites, respectively. To neutralize the residual positive charge of the layer, anions intercalate between the layers. Nitrate anions intercalate between the layers in the case discussed here.

Natural minerals contain more than one metal, which has led to identification of several layered hydroxide slats containing two different divalent metals. The resulting compounds are denominated double hydroxide salts (DHSs). They have the generic formula M^a^_1−x_M^b^_x_(OH)_2−x_(A^n−^)_x/n_·yH_2_O, where M^a^ and M^b^ are two different divalent cations, and A is the counter anion with charge n^−^. Aurichalcite ((Zn,Cu)_5_(CO_3_)_2_(OH)_6_), Haydeeite (Cu_3_Mg(OH)_6_Cl_2_) and Kapelassite (Cu_3_Zn(OH)_6_Cl_2_), among others [45], are examples of these structures.

Less frequently, layered hydroxide salts with three different metals have been reported; e.g., Loseyite (Mn,Zn,Mg)_4_Zn_3_(CO_3_)_2_(OH)_10_ or Sclarite (Mn,Zn,Mg)_4_Zn_3_(CO_3_)_2_(OH)_10_ [46,47].

## 2. LDH and LHS as Support for Metalloporphyrin Immobilization

### 2.1. Layered Double Hydroxides (LDHs)

Natural or synthetic layered compounds can immobilize a great variety of charged species, leading to several applications. These compounds are commonly used as ion exchangers [48], but they can also be employed in catalysis [49] and in the biological and medical fields [50], not to mention their use as flame retardants [51].

MP immobilization or even intercalation into LDHs has been known for over 20 years, as attested by the review of Bedioui published in 1995 [11]. In 2012, Demel and Lang published a short review dedicated to MP immobilization and application of the resulting material in catalysis [30].

To discuss MP immobilization on LDHs and the catalytic activity of the final materials, it is worth mentioning the work of Wypych *et al.* [52] published in 2003, which reported immobilization of an anionic iron (III) porphyrin (FeP) through LDH exfoliation processes. Based on the XRD data, the FeP was probably located on the surface of the exfoliated and restacked layered crystals. Later, the first investigation into the catalytic activity of FePs immobilized on exfoliated LDHs as heterogeneous catalysts was reported [53]. These materials catalyzed hydroxylation reactions selectively and efficiently. Their reuse afforded higher yields than the parent fresh catalyst probably because the LDH crystals re-accommodated and exposed the active FeP catalytic sites [53].

In the last years, different strategies have helped to create new catalysts involving MP immobilized on different LDHs. In 2013, He and co-workers developed a new catalyst by encapsulating the MnP [Mn(TPP)OAc] into Zn/Al–LDHs (an LDH prepared with Zn^2+^ and Al^3+^) intercalated with dodecyl sulfonate. These authors designed and prepared this solid aiming to obtain a structure that resembled the structure expected for a bilayer of naturally occurring phospholipids [54]. The catalytic activity of the resulting catalyst in the oxidation of a series of alkenes including cyclohexene, heptylene, phenylethylene, 3-methyl-3-buten-1-ol, ethyl cinnamate, and chalcone afforded superior results as compared to the parent compound in solution (homogeneous reactions), which indicated that this material had promising activity for alkene oxidation. These immobilization strategies opened the possibility to immobilize neutral as well as anionic MPs between LDH layers.

Recently, Jaina *et al.* [55] published a work where they described the synthesis of a magnetically separable LDH named Mg/Al-LDH@Fe_3_O_4_. The authors used this solid to immobilize a cobalt phthalocyanine catalyst. The resulting material effectively catalyzed the oxidation of mercaptans by molecular oxygen in aqueous medium. An external magnetic field promoted easy recovery of this catalyst from the reaction medium [55]. Facile catalyst recovery and reuse allied with the use of molecular oxygen and water as solvent qualified this solid as an interesting catalyst for green processes.

Nakagaki and co-workers [56] recently reported immobilization/intercalation of an FeP into exfoliated macroporous LDH. Oxidation of two model substrates, (*Z*)-cyclooctene and cyclohexane, by PhIO in the presence of this material proved that the immobilized FeP not only was a potential catalyst for heterogeneous processes but also had promising technological applications.

A second-generation MnP intercalated into the interlayer space of different LDHs containing Mg^2+^ or Ni^2+^ and Al^3+^ by the exfoliation/restacking approach, using organic groups as building blocks, efficiently and selectively catalyzed cyclohexene epoxidation by molecular oxygen in the presence of isobutylaldehyde as co-reductant [57]. The reused catalyst afforded product yields that resembled the yield achieved with the fresh catalyst and that were higher than the yield obtained during homogeneous catalysis (MP catalyst in solution).

More complex structures like iron or manganese glycol metalloporphyrin immobilized on LDHs have also been investigated as catalysts for hydroxylation and epoxidation reactions by iodosylbenzene. The immobilized MPs provided good catalytic yield in the epoxidation reaction, and they selectively catalyzed cyclohexane oxidation. The solids were used in at least three catalytic cycles in the case of epoxidation reactions. The reusability and stability of the MP-LDH catalysts pointed to a promising and economically viable process [4].

The use of LDHs as support for MPs has applications in other fields. Bearing in mind that free-base porphyrins can act as photosensitizers in allylic oxidation, researchers have investigated the photocatalytic activity of the [Fe(TPFPP)Cl]/Mg/Al-LDH system for aerobic alkene epoxidation under visible-light irradiation. The catalyst was selective for 1,2-epoxycyclohexane during photocatalytic aerobic cyclohexene epoxidation. The authors concluded that [Fe(TPFPP)Cl]/Mg/Al-LDH was an effective photocatalyst for the oxidation of various alkenes [58].

Natural protein immobilization is another research field that uses the versatile LDHs associated with tetrapyrrolic macrocycles. Different techniques such as XRD (powder), UVVIS, and fluorescence spectroscopies have aided understanding of the role that ionic liquid plays in the interaction between LDHs and hemoglobin (Hb) upon Hb immobilization on Zn_2_Al-LDH modified with hydroxyl-functionalized ionic liquid by the co-precipitation or the adsorption method. Investigation of the electrocatalytic activity of the immobilized Hb by cyclic voltammetry showed that co-precipitation was apparently more effective for enzyme immobilization, and that the resulting materials constituted electrochemical biosensors [59].

In order to facilitate the lecture, Table 1 summarizes the main MP immobilized on LDH and discussed above.

#### 2.1.1. Other Macrocycles Immobilized in LDH

Macrocyclic compounds are among the species that can be immobilized on LDH supports. In recent decades, the number of investigations into these synthetic compounds has increased and improved our understanding of their coordination chemistry. Because of their relative stiffness and variety, these compounds can form complexes with different metals, which leads to a huge variety of applications that range from biological models to sophisticated catalytic applications.

**Figure 5:**
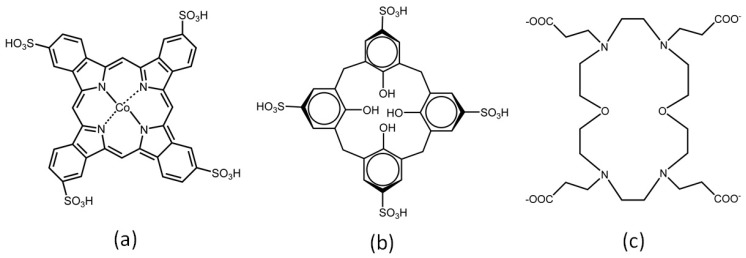
Structure of: (**a**) Co(II) tetrasulfophthalocyanine; (**b**) Carboxyethyl substituted azacrown ether derivative (CSAE); (**c**) *p*-sulfonated calixarene.

Various macrocyclic complexes can interact with LDHs, especially phthalocyanines and porphyrins. More recently, interaction of anionic crown ethers and calixarene compounds (Figure 5) with LDHs has been described [60,61,62,63,64,65,66,67,68,69,70,71].

Pinnavaia *et al.* [60] authored the first paper on a macrocyclic complex interlayered in an LDH and reported on a promising catalyst to oxidize 2,6-di-tert-butylphenol: a catalyst based on heterogenization of a tetra anionic Co(II) tetrasulfophthalocyanine [CoPcTs]^4−^ (Figure 5a) intercalated into a Mg/Al LDH at M^2+^/M^3+^ molar ratio of 2:1 and 4:1. The turnover frequency of the immobilized compounds was twice as great as the turnover frequency of the complex [CoPcTs] in solution, which indicated that the substrate had easier access to the active center. The authors also concluded that heterogenization inhibited Co(II) phthalocyanine dimerization [60]. Several other authors have evaluated phthalocyanines as catalysts for countless catalytic reactions such as oxidation of 1-decanethiol [60], 2,6-di-*tert*-butylphenol (to the corresponding diphenoquine) [61], and mercaptan [62] as well as photocatalytic methylene blue decoloration [63].

Carraro *et al.* developed various solids based on either anionic or cationic copper(II) phthalocyanine intercalated into clay minerals and LDH [64]. Basal distance results obtained by X-ray diffraction of the resulting LDH materials evidenced that the phthalocyanine intercalated between the LDH layers. The prepared solid was based on a copper(II) phthalocyaninetetrasulfonic acid tetrasodium salt (CuPcTs) intercalated into LDH, where the immobilized metallophthalocyanine was perpendicular to the LDH layers. After publication of this paper, many other studies involving different metallophthalocyanines intercalated into LDHs were prepared to ascertain their mode of interaction with the LDH. Here, the studies reported by Ukrainczyk and co-workers are worthy of mention [65,66].

Macrocycles derived from the crown ether family are also interesting to intercalate into LDHs. Ma *et al.* intercalated a carboxyethyl-substituted azacrown ether derivative (CSAE) (Figure 5b) into a Mg/Al-LDH by exchanging the crown ether with the intercalated nitrate anions. These authors aimed to understand how the CSAE accommodated between the LDH layers, prepared under different conditions [67]. Although the LDH contained co-intercalated carbonate anions, it was possible to predict the arrangement modes of the intercalated species. Because the azacrown ether molecules were flexible, they inclined and the crown could assume distinct orientation modes.

Ma *et al.* also investigated staging formation after swelling of the intercalated LDH in formamide. The structure was restored in the presence of the macrocyclic tetraazacrown ether carboxylic acid derivative (TECA) [68]. Similarly, a procedure was developed for direct “one-step” intercalation of the macrocyclic tetraazacrown ether carboxylic acid derivative (TECA) into the Mg/Al-LDH interlayer [69]. In both cases [68,69], TECA co-intercalated with nitrate or carbonate anions, depending on the synthesis procedure.

Sasaki and co-workers synthesized *p*-sulfonated calix [4 (CS4) and 6 (CS6)] arene (Figure 5c) intercalated into LDHs and studied the ability of the resulting material to adsorb benzyl alcohol (BA) and *p*-nitrophenol (NP) [70,71]. The authors varied the chemical composition of the LDH support by using different divalent metals (Mg^2+^ and Zn^2+^) and keeping Al^3+^ as the trivalent metal; the M^2+^/M^3+^ molar ratio was 3:1. The authors also varied the number of calixarene ring units (four and six phenol units). For CS6 (six phenol rings), there were no differences in the interaction mode of calixarene with the LDH layers; that is, the immobilized compound was perpendicular to the LDH regardless of the presence of Mg^2+^ or Zn^2+^. However, for CS4 (four phenol rings), there were two immobilization modes, which depended on the LDH composition. The X-ray diffraction patterns showed that CS4 intercalated vertically and horizontally relative to the Mg/Al-LDH and Zn/Al-LDH layer plane, respectively (d_003_ = 1.33 nm and 1.61 nm, respectively). The distinct CS4 orientations were due to the different pH values used during the synthesis (pH = 10 and 7 for Mg/Al-LDH and Zn/Al-LDH, respectively), which ionized a different number of OH groups in CS4 (p*K*_a1_ = 3.08 and p*K*_a2–4_ > 11). Different CS4 orientation modes strongly influenced BA and NP adsorption on the surface of LDH crystals. BA adsorption was 2.10 and 0.22 (BA/CS4 molar ratio) for CS4 intercalated into Zn/Al-LDH and Mg/Al-LDH, respectively, and NP adsorption was 4.50 and 0.76 (NP/CS4 molar ratio) for CS4 intercalated into Zn/Al-LDH and Mg/Al-LDH, respectively. Therefore, CS4 intercalated into Zn/Al-LDH (where CS4 was parallel to the layers) adsorbed larger quantities of BA and NP than CS4 intercalated into Mg/Al-LDH (where CS4 was perpendicular to the layers).

### 2.2. Layered Hydroxide Salts (LHSs)

Despite reports on the synthesis of some LHSs in the 1970s [72,73], only recently did LHSs as support and LHSs for other applications gain attention [43,74,75,76]. LHSs can serve as support for anions derived from mono- and dicarboxylic acids [77], sodium dodecyl sulfate [75], different dyes [78], and palladium porphyrins [79]. Some authors investigated LHS surfaces [74] and the use of LHSs as catalyst for esterification of free fatty acids [80].

**Figure 6:**
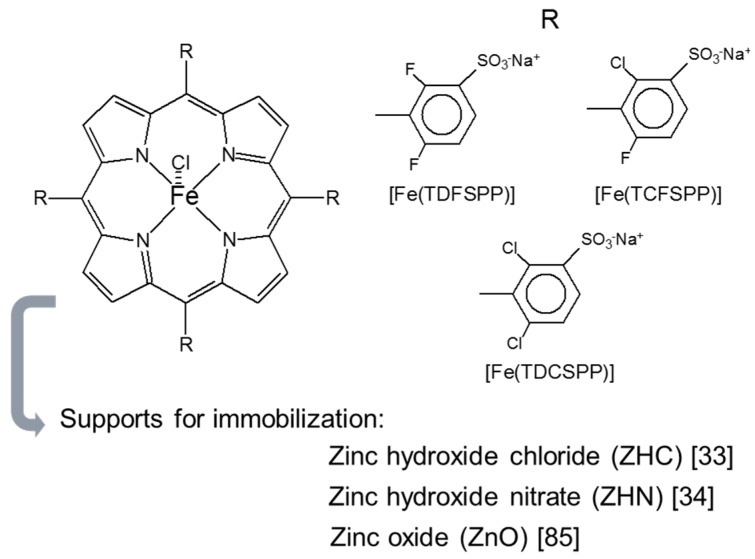
Structure of the anionic iron(III) porphyrins (FeP) employed in references [33,34,85]. [Fe(TDFSPP)Na_4_]Cl = [tetrasodium-5,10,15,20-tetrakis(2,6-difluoro-3-sulfonatophenyl)porphyrinate iron(III)] chloride; [Fe(TCFSPP)Na_4_]Cl = [tetrasodium-5,10,15,20-tetrakis (2-chloro-6-fluoro-3-sulfonatophenyl)porphyrinate iron(III)] chloride, and [Fe(TDCSPP)Na_4_]Cl = [tetrasodium-5,10,15,20-tetrakis (2,6-dichloro-3-sulfonatophenyl) porphyrinate iron(III)] chloride.

Our research group first reported on LHSs as supports to immobilize MPs for use in catalyzed heterogeneous oxidation processes in 2010 [34]. In this work, a family of second-generation anionic FePs (Figure 6) were immobilized on the surface of zinc hydroxide nitrate (ZHN) and investigated as oxidation catalysts for heterogeneous cyclooctene, cyclohexane, and *n*-heptane oxidation [34]. Besides the excellent yields that the catalysts provided during cyclooctene and *n*-heptane oxidation, they were surprisingly selective for cyclohexanone instead of the alcohol product. During cyclohexane oxidation, the catalyst FeTDCSPP-ZHN (FeDC-ZHN) afforded up to 70% cyclohexanone and only 2% cyclohexanol after one hour of reaction [34], this behavior contrasted with the classic mechanism of oxidation catalyzed by FePs in solution (homogeneous catalysis), which are frequently selective for cyclohexanol [81,82,83,84] and act via the catalytic species ferryl porphyrin π-cation radical Fe^IV^(O)P^●+^ [5,10,34,81,82]. In FeDC-ZHN, the FeP probably interacted with the ZHN surface, to create an ambience that favored the radical oxidation mechanism instead of the classic oxidation mechanism [17]. In this same work by our group [34], the FeP-ZHN catalysts were selective for the alcohols instead of the ketone during *n*-heptane oxidation. These results strongly suggested that the structure of the substrate and the FeP immobilization mode tuned the catalytic results. The structure of *n*-heptane is linear, whereas the cyclic structure of cyclohexane can acquire the “boat” and “chair” configurations [19]. This culminated in distinct selectivity results for the two substrates even though the catalyst was the same.

The same family of FePs (Figure 6) was also immobilized on layered zinc hydroxide chloride (ZHC) [33]. Replacing the exchangeable nitrate ion with the chloride ion grafted in the layer composition elicited a different catalytic activity. The FeP immobilization degree in LHSs changed (immobilization degree is the percentage of FeP immobilized on the solid support relative to the mass of FeP used during the immobilization procedure). For the same immobilization reaction conditions, the immobilization degree of the three FePs on ZHN was near 100% [34], whereas the immobilization degree was approximately 30% [33] in the ZHC support. This difference was attributed to non-effective coordination of nitrate to the ZHN layers [43]. The anionic FeP substituted the free nitrate ion located between the layers and on the ZHN solid surface [34,79]. In the case of the ZHC support, the chloride ions were directly coordinated to the layers [43] and could not be exchanged with the anionic complex. This left only the positive charges on the crystal edges to interact with the FePs [33]. FeP immobilization on different LHS sites directly influenced the oxidation catalysis results. Catalysts immobilized on ZHN and ZHC were selective for cyclohexanone and cyclohexanol during cyclohexane oxidation, respectively [33], which meant that the reaction followed the classic route expected for FePs in solution in the latter case [10,17,82]. Indeed, FeTDCSPP-ZHC (FeDC-ZHC) afforded 23% cyclohexanol and 2% cyclohexanone yields [33] *vs.* 70% cyclohexanone and 2% cyclohexanol achieved in the presence of FeTDCSPP-ZHN in the same reaction conditions. The reaction conducted in the presence of the FeP immobilized on ZHN followed a radical mechanism, and the total product yield (ketone + alcohol) was higher than the total product yield obtained in the case of the FeP immobilized on ZHC. These examples attested that MPs are versatile catalysts and point to the importance of the support when preparing a solid catalyst for heterogeneous processes.

Demel and Lang published a short review about layered hydroxide-porphyrin hybrid materials [30] where they summarized findings for some systems involving LDHs and LHSs containing intercalated anionic MPs or phthalocyanines. The authors discussed some aspects like synthetic strategies for the intercalated compounds, structural aspects based on characterization techniques, photo functional applications, and use in catalysis. To the best of our knowledge, there has been no other work on MPs immobilized on LHSs as catalysts for oxidation reactions since this review. However, this is a very interesting research field: many LHSs, with diverse metallic ions (e.g., Cu^2+^, Co^2+^, and Ni^2+^) and interlayer anions (e.g., Cl^−^, CO_3_^2−^, SO_4_^2−^, and organic anions with different chain lengths), can serve as support for MP immobilization. Some of the resulting catalysts could even elicit unusual selectivity for cyclohexane oxidation, as observed when ZHN was the support [34].

Despite the rare use of LHSs as support for MPs and their potential application as oxidation catalysts, an interesting property of LHSs has been investigated for MP immobilization: the *in situ* generation of oxides from the layered structure [85,86,87,88,89].

#### 2.2.1. Oxides Derived from LHSs

LHSs can be decomposed into the respective oxide by thermal treatment. For example, treatment of zinc hydroxide carbonate at 300 °C [86], zinc hydroxide nitrate at 400 °C [87], and zinc hydroxide sulfate at 900 °C [88] generates the respective ZnO.

Hydrothermal treatment can also transform LHS into the corresponding oxide [85,89] by keeping the LHS suspension in water in reflux at 100 °C [85] or in an autoclave at 140 °C [89]. Another way to obtain the respective oxide is to increase the pH during the LHS synthesis the LHS does not separate from the reaction mixture, and the oxide emerges at pH higher than 8 [43,79]. Musić *et al.* employed 8 mol·L^−1^ NaOH solution to obtain ZnO directly from a 1 mol·L^−1^ Zn(NO_3_)_2_ solution [90]. The method used to obtain the oxide depends on the research objective.

The oxide obtained from LHSs has a high degree of purity, and its crystals retain the morphology of the initial LHS (topotactic growth) [43,88]. Another advantage is that the methods that produce these oxides are easy to accomplish.

In 2011, Huang and co-workers published a work where they immobilized a cobalt tetra(4-hydroxyl)phenylporphyrin [Co(THPP)] on ZnO obtained from zinc hydroxide sulfate [24]. To synthesize the support, the researchers used a zinc sulfate heptahydrate solution, which provided the metal and NaOH (as the source of hydroxide ions). The authors then immobilized [Co(THPP)] on the obtained solid, and the final solid was dried at 160 °C for 5 h [24]. The solid material selectively catalyzed toluene oxidation by O_2_ to benzaldehyde and benzylalcohol. The [Co(THPP)]/ZnO solid was a better catalyst than [Co(THPP)] in homogeneous solution due to a cooperative association between the support and the MP [24]. As verified in the case of FePs immobilized on ZHN [34], the support was not innocent or inert during the catalytic reactions.

In 2013, our research group used ZnO as support to immobilize the same FeP family that our group had immobilized on ZHN [34] and ZHC [33]. ZHN hydrothermal decomposition furnished ZnO [85]. Addition of each FeP (Figure 6) was to a ZHN suspension in water, and 5 h under stirring and reflux generated ZnO containing the immobilized complex [85]. Changing the LHS to the oxide caused collapse of the layered structure and loss of the tetrahedral zinc sites, to give a non-corrugated ZnO surface. Anionic MP immobilization on the ZnO surface was possible because residual positive charges existed on the oxide surface in the pH employed during the immobilization process [43]. Loss of the ZHN corrugated surface re-established the usual FeP behavior for the FePs immobilized on ZnO; that is, selectivity for cyclohexanol during cyclohexane oxidation [85]. In the case of FeTDCPP-ZnO, the cyclohexanol and cyclohexanone yields were 22% and 3%, respectively, which resembled the results achieved for FeTDCSPP-ZHC (cyclohexanol and cyclohexanone yields of 23% and 2%, respectively) but contrasted with the results obtained for FeTDCSPP-ZHN (cyclohexanol and cyclohexanone yields of 2% and 70%, respectively). These data corroborated the fact that the environment created around the FeP in ZHN was unique and led to the radical route during cyclohexane oxidation. Decomposition of the corrugated structure made the reaction mechanism return to the classic route [17,34,85].

In 2015, Huang and co-workers immobilized an iron(III)) tetrakis(pentafluorophenyl)porphyrin [Fe(TPFPP)] onto ZnO and studied the catalytic activity of this solid during cyclohexane oxidation [91]. These authors used the same procedure employed in our work of 2011 [24] to obtain ZnO and to immobilize [Fe(TPFPP)] on the support. The authors detected a blue shift in the Soret band (from 416 nm to 405 nm) upon [Fe(TPFPP)] immobilization on ZnO. This behavior was attributed to face-to-face/parallel immobilization of the FeP on the ZnO surface, which led the negative fluorine atoms and the positive ZnO surface to interact and give a more planar [Fe(TPFPP)] structure. The authors used [Fe(TFPP)]-ZnO to catalyze cyclohexane oxidation by O_2_ in an autoclave at 150 °C; they did not use solvent or co-catalysts [91]. At 150 °C, 1.0 mg of [Fe(TPFPP)] in ZnO gave product yield of 22.5%, which was higher than the yield obtained with 1.0 mg of non-immobilized [Fe(TPFPP)]: 15.0%. According to the authors, the higher reaction yield resulted from the cooperative effect between the FeP and the support, probably FeP coordination to ZnO, as explained in the former work by our group [24]. Huang and co-workers [91] also proposed that a peroxide species arose during catalysis and underwent rapid conversion to the desired products.

Still in 2015, Xie *et al.* immobilized a [*trans*-CoD(*p*-Cl)PP] on various supports [25] such as boehmite, ZrO_2_, MCM, ZnO, and others. These authors obtained ZnO from a zinc sulfate heptahydrate aqueous solution and NH_4_OH (as the hydroxide source). The authors tested cyclohexane oxidation by O_2_ in an autoclave, without solvent or co-catalyst, in a procedure resembling the procedure described by Huang *et al.* [91]. The researchers investigated how pressure, temperature, and time affected the catalytic reaction [25]. They reported that the best support was ZnO, which afforded good substrate conversion, high turnover number, and selectivity.

Finally, Li and co-workers [92] used a Zn(CH_3_COO)_2_ aqueous solution added with NaOH aqueous solution, treated in an autoclave at 120 °C, to generate ZnO microrods. The authors immobilized a hetero aggregate of porphyrins (tetrakis(4-trimethylaminophenyl) porphyrin [H_2_(TAPPI)] and tetrakis(4-sulfonatophenyl) porphyrin cobalt(II) [Co(TPPS)]) on this support and employed it in the photodegradation of the rhodamine B dye with good results [92]. Therefore, ZnO obtained from LHSs is a versatile support.

## 3. Influence of the LDH Composition on the MnP Immobilization Rates and Catalytic Performance: New Results

Motivated by the rich chemistry of MP immobilization on layered compounds and its implication for the efficiency and selectivity of the obtained solids, our research group has studied how the LDH composition impacts the immobilization rates and catalytic performance in cyclooctene oxidation of [5,10,15,20-tetrakis(2,6-difluoro-3-sulfonatephenyl porphyrinate) manganese(III)] acetate (MnP) (the parent FeP is illustrated in Figure 6).

First, we synthesized different LDH solids by obtaining LDHs from magnesium or zinc salts (M^2+^) and aluminum (M^3+^) at different nominal molar ratios (M^2+^/M^3+^ = 2:1, 3:1 or 4:1), with different interlayer anions (CO_3_^2−^ and NO_3_^−^). The co-precipitation method with increasing pH yielded LDHs containing intercalated carbonate or nitrate [93]. Table 2 shows the nomenclature used for the obtained solids.

**Figure 7:**
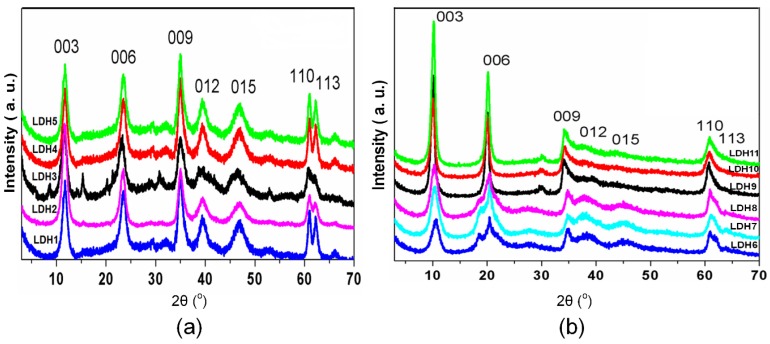
XRD patterns (powder) of the prepared LDHs intercalated with: (**a**) CO_3_^2−^; (**b**) NO_3_^−^.

X-ray powder diffraction (Figure 7) aided characterization of the solids. The calculated basal distances agreed with literature data on this class of compounds and were typical of an LDH solid bearing intercalated carbonate (~7.65 Å) (Figure 7a) or nitrate (~8.79 Å) (Figure 7b) ions [94,95,96]. Recently, the structure of LDHs have been obtained also by X-ray [97] or electron diffraction methods [98].

The cell parameters “a” referred to the distance between the metal ions present in the LDH layer. Table 2 lists the values for this parameter and revealed an increasing trend higher M^2+^/M^3+^ ratio increased the ionic radius of each M^2+^ (the ionic radius of Mg^2+^ and Zn^2+^ is 0.74 Ǻ and 0.66 Ǻ, respectively). 

Infrared analyses of the synthesized LDHs (Supplementary Data, Figure S1) evidenced the typical vibrational bands profile of these materials. The band at 3434 cm^−1^ was related to stretching of the O–H bond in the hydroxyl groups of the layers and the co-intercalated water molecules. The band at 1625 cm^−1^ was due to angular deformation of water molecules. The bands in the region below 600 cm^−1^ were characteristic of metal-oxygen (M–O) stretching. The spectra of the LDHX solids (X = 1–5) displayed the typical bands of carbonate ions at 1366 cm^−1^ and 1636 cm^−1^. The solids also featured a band at 1382 cm^−1^, characteristic of nitrate ions, which was more pronounced in the spectrum of LDH4. This small contamination with nitrate ions probably resulted from the starting chemicals (nitrate salts).

In the spectra of the LDHX solids (X = 6–11), bands ascribed to nitrate ion appeared at 1382 cm^−1^ and 1023 cm^−1^. In the same spectra, a shoulder at 1366 cm^−1^ indicated that these compounds also contained traces of carbonate ions [94,96].

Thermogravimetric analysis (TGA) helped to estimate the water content (physisorbed and intercalated) and the chemical composition of the solids based on the residual oxides content (Supplementary Data, Figure S2) (Table 3) [99].

Table 4 summarizes the solids resulting from MnP immobilization on the different LDH solids (named MnP-LDHX, where MnP refers to [Mn(TDFSPP)] and X refers to the prepared LDH).

**Figure 8:**
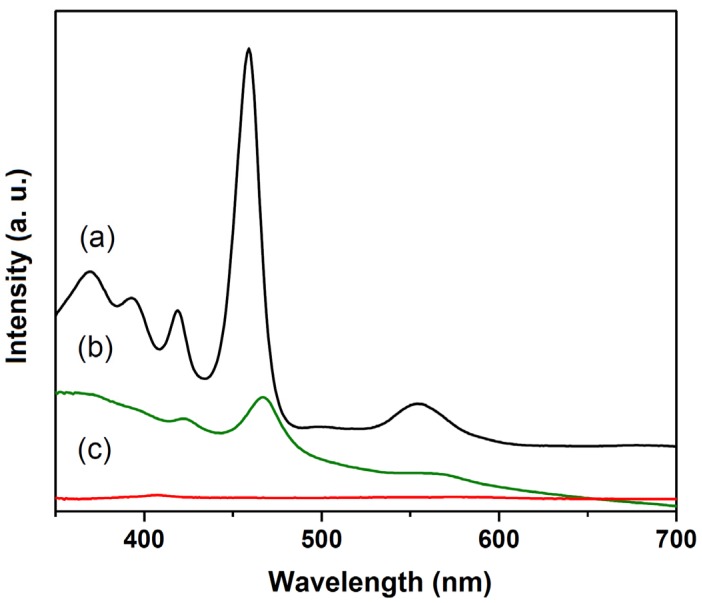
UVVIS spectra: (**a**) Methanolic MnP solution; (**b**) Solid Nujol emulsion of MnP-LDH3; (**c**) Supernatant collected after the immobilization process.

Figure 8 brings the UVVIS results obtained for the prepared solids. Figure 8b depicts the UVVIS spectrum (in Nujol mull) of immobilized MnP-LDH3. The spectra displayed the typical MnP Soret band near 463 nm [84], confirming MnP immobilization on the solid. Lack of changes in the basal distance, as revealed by X-ray analysis (data not shown), indicated MnP surface immobilization and excluded MnP intercalation between the LDH layers. All the prepared solids presented the same behavior.

Figure 8a depicts the UVVIS spectrum of the methanolic MnP solution. The Soret band appeared at 457 nm. The UVVIS spectra of the supernatant collected after the immobilization process (Figure 8c) confirmed MnP immobilization close to 100% for all the prepared solids.

We excluded the interaction of the MnP with the layered crystal edges, which was negligible in terms of quantity. Then, we attempted to understand the mode of MnP immobilization on the surface of the LDH support by performing some calculations.

Considering the ideal M^+2^/M^+3^ molar ratios used in the synthesis, where the M^+3^ concentration led to a proportional concentration of positive charges on the surface of the layered crystals, we expected that the tetranegatively charged MnP would establish different modes of interaction.

**Figure 9:**
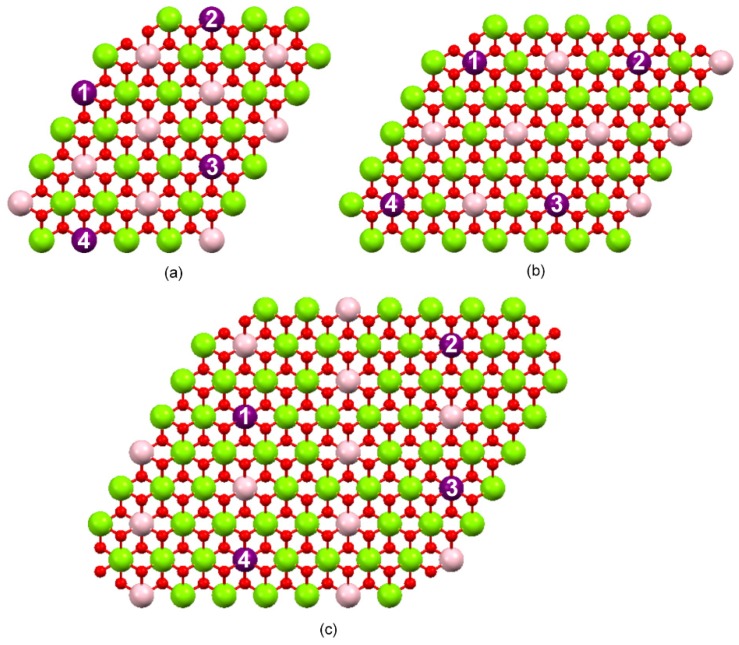
Schematic representation of the structure of the LDH prepared at Mn^2+^/M^3+^ molar ratio of: (**a**) 2:1; (**b**) 3:1; (**c**) 4:1. The pink and purple balls represent the species M^3+^, the green balls represent the species M^2+^ and the red balls represent the oxygen atoms from the hydroxyl ions. Figure built with the aid of the Mercury^®^ free software.

Because both adjacent layers should share the charges, the positive charge on the layer surface should be half of the value and the distances between charges should be twice the distance between the M^+3^ cations. This calculation was based on the values found for the cell “a” parameter (Table 2), which measures the distance between the metal ions present in the layers (Table 5). As for d_1,2_, it represents the distance between the numbered purple balls labeled 1 and 2 in Figure 9. The same representation applies for the distances between purple balls labeled 2 and 3 (d_2,3_), 3 and 4 (d_3,4_), 4 and 1 (d_4,1_), 1 and 3 (d_1,3_) and 2 and 4 (d_2,4_).

**Figure 10:**
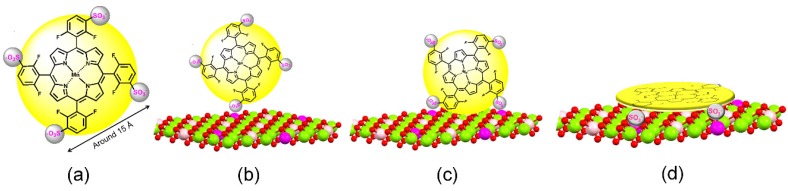
Schematic representation of the possible modes of MnP (yellow ball interaction with the LDH surface: (**a**) MnP; (**b**) MnP interacts only via one negative charge; (**c**) via two negative charges; (**d**) via four negative charges.

Software *Hyperchem^®^* helped to determine the distances between the MnP negative charges (SO_3_^−^) (Figure 10a) by optimization of the MnP structure. Figure 10b–d show a schematic representation of the possible modes of MnP interaction with the layered crystal surface.

The first proposal (Figure 10d) represents interaction of four negative charges in the MnP with four positive charges in the support layer. For the MnP to immobilize on the LDH in this way, the calculated distances between the positive charges present in the support layer must precisely match the distances between the four MnP negative charges. This limitation allowed us to exclude this arrangement from the formulated hypothesis.

In the second representation (Figure 10c), two MnP negative charges interact with LDH. Values described in Table 5 were based on the distance between two LDH positive charges and on the distance between the MnP SO_3_^−^ substituents (negative charges). Analysis of the values of positive charges in the LDH layers presented in Table 5 (indicated by *) suggested that this kind of interaction (by two charges) was possible for MnP immobilization on the LDH prepared at only M^2+^/M^3+^ molar ratio of 4:1.

Figure 10b corresponds to yet another proposal for MnP immobilization on the LDH-MnP would be in contact with the LDH surface just via one negative charge. This is probably the most feasible proposal because it only depends on the approach of one opposite charge and dismisses the need to match the distances between the MnP negative charges and the LDH surface layer positive charges.

We evaluated the prepared solids as catalysts for cyclooctene oxidation in an attempt to understand how the M^+2^/M^+3^ molar ratio, the type of intercalated anion (single-charged nitrate and double-charged carbonate), and the mode of MnP immobilization influenced the MnP catalytic activity. Cyclooctene was selected because it is commonly employed as substrate during investigation into the catalytic activity of MPs [16,17,83,100,101].

**Figure 11:**
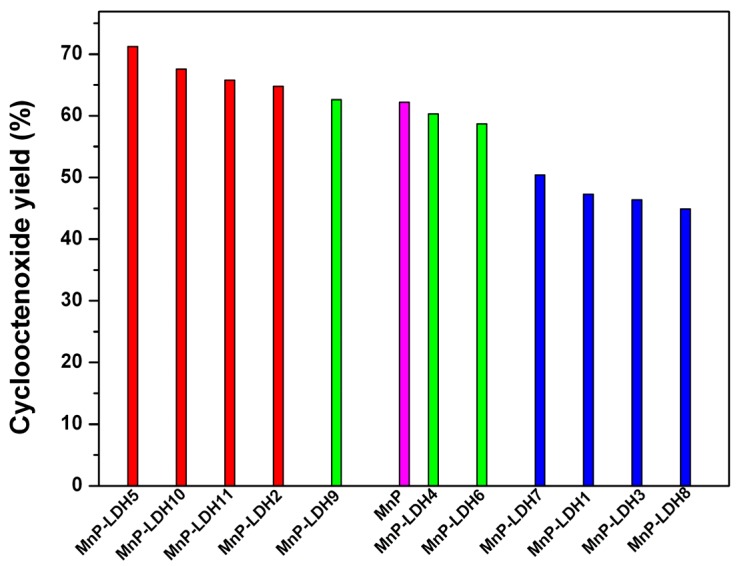
Comparison of the product yield obtained with MnP during homogeneous cyclooctene epoxidation (magenta bar) and of the product yield obtained for each of the prepared solid catalysts (MnP-LDHX). The yield attributed to the control reaction has been subtracted in all cases.

Although all the solid catalysts contained the same MnP, they afforded distinct catalytic results (Table 6). Based on Table 6, rationalizing the effect of the support on product yield was not easy. However, analysis of the last column of Table 6, which provided the epoxide yields by excluding the yield obtained for the control reactions (reactions performed with pure LDH without immobilized MnP, Table 6, runs 13–23) revealed some interesting trends in the catalytic behavior of the MnP immobilized on the prepared LDHs. Figure 11 represents these differences and trends better.

MnP immobilization on LDH5, LDH10, LDH11, and LDH2 (red bars in Figure 11) produced solid catalysts that gave higher product yields than the same MnP during homogeneous catalysis (magenta bar in Figure 11). Most of these prepared LDHs contained high Zn/Al molar ratio. The intercalated anion did not seem to affect the catalytic results. The only exception to this trend was LDH2, which corresponded to a Mg/Al-LDH but had the same M^2+^/M^3+^ molar ratio as LDH5 (3:1). Indeed, the catalytic result achieved with MnP-LDH2 was on the borderline between the red and the green group in Figure 11. All the solids in the red group gave rise to higher catalytic performance as compared to the MnP in homogeneous solution.

The group of green bars in Figure 11 represented the solid catalysts that gave product yield similar to the yield obtained with the same MnP during homogeneous catalysis, namely MnP-LDH9 (Zn/Al 2:1), MnP LDH4 (Zn/Al 2:1), and, at the borderline between the green and blue groups, MnP-LDH6 (Mg/Al 2:1). The catalysts in the green group showed that MnP immobilization did not translate into the catalytic advantage observed in the case of the catalysts in the red group. The only advantage of MnP immobilization in the green group was prevention of MnP catalytic activity loss and possibility of catalyst recovery and reuse after the first cycle.

Finally, the blue group corresponded to the solid catalysts that afforded lower epoxidation yield than the yield furnished by the MnP during homogeneous catalysis, more specifically MnP-LDH7 (Mg/Al 3:1), MnP-LDH1 (Mg/Al 2:1), MnP-LDH3 (Mg/Al 4:1), and MnP-LDH8 (Mg/Al 4:1). All the LDHs in this group contained Mg^2+^ in their compositions, showing that this ion abated the MnP catalytic performance irrespective of the intercalated anion and of the M^2+^/M^3+^ molar ratio.

These results suggested that the characteristics of the MnP/support catalytic system depended on the LDH and on the kind of interaction established between the MnP and the support. At a first glance, one might think that the access of reagents to the MnP was a function of the divalent metal present in the LDH.

M^2+^/M^3+^ molar ratios of 3:1 in the LDH seemed to favor the MnP catalytic activity. This may have been related to the way that the porphyrin attached to the LDH surface and showed that changes in support composition may have affected the approach of the substrate to the catalytically active center. This effect was more evident for Zn/Al-LDH.

In general, LDH containing Zn^2+^ ions furnished the best catalytic results. The effect of zinc *versus* magnesium is not easy to explain, and studies on how different LDH compositions impact the catalytic oxidation results are rare.

Zhang *et al.* recently prepared gold nanoclusters (AuNCs) supported on M_3_Al-LDHs (M = Mg^2+^, Ni^2+^, Co^2+^) and used these solids to catalyze aerobic 1-phenylethanol oxidation under solvent-free conditions [102]. The AuNCs/M_3_Al-LDH (where M = Ni^2+^ or Co^2+^) catalysts presented higher alcohol oxidation activity than AuNCs/Mg_3_Al-LDH. Many factors contributed to these results: (i) generation of a larger amount of hydroxyl groups in the presence of Ni^2+^ and Co^2+^ during preparation of the M_3_Al-LDH support, which implied that Brønsted basic sites existed in the AuNCs/M_3_Al-LDH catalysts; (ii) high acidity of the LDH surface in the presence of AuNCs; (iii) critical effect of the metal components in the LDH supports on the electronic structure of AuNCs; (iv) more negatively charged AuNCs in AuNCs/Ni_3_Al-LDH and AuNCs/Co_3_Al-LDH; (v) larger electronegativity of the transition metals Ni and Co as compared to Mg, which may have facilitated electron transfer from the support to the AuNCs, resulting in more electron-rich Au cores in AuNCs/Ni_3_Al-LDH and AuNCs/Co_3_Al-LDH than in AuNCs/Mg_3_Al-LDH. The authors claimed that all these factors elicited a synergistic effect between the LDH and AuNCs, resulting in better catalytic performance for AuNCs/Ni_3_Al-LDH and AuNCs/Co_3_Al-LDH during the oxidation reaction.

In another recent example, Sun *et al.* [103] prepared a structured catalyst by immobilizing a cobalt phthalocyanine tetrasulfonate (CoPcS) onto multimetallic oxides of the Mg,Ni/Al-MMO type (obtained by thermal decomposition of Mg,Ni/Al-LDH) with tunable basicity. Their purpose was to enhance the synergistic effect between the active center (CoPcS) and the basicity of the support to the maximal extent; they used the Mercaptan Sweetening catalytic reaction via oxidation of 1-octanethiol to the corresponding disulfide as model reaction [101]. Changes in the relative content of Mg^2+^ and Ni^2+^ in the LDH precursor used to prepare the support Mg,Ni/Al-MMO by thermal decomposition conveniently tuned the basicity and the number of moderately basic sites in the solid designated CoPcS/Mg,Ni/Al-MMO. Alterations in the Mg/Ni molar ratios increased the conversion value: Mg/Ni molar ratio of 1.16 gave maximum conversion (93%). The authors claimed that the synergistic effect between the oxidation center (CoPcS) and the large number of moderate basic sites (0.49 mmol/g, determined by CO_2_-TPD) present in the catalyst support was optimal at this Mg/Ni molar ratio. Thereafter, conversion values decreased. The authors concluded that the intrinsic mechanism of the synergistic should be rather complicated, which is the reason why it is under investigation in their research lab.

Together, these few examples corroborated our preliminary results on the active influence of the catalyst support on the oxidative catalytic activity of the MnP during cyclooctene oxidation.

The possible effect of the water present in the interlayer space and outer surface of the prepared LDHs should not be ignored. Percentage H_2_O mass loss in the solid LDHs without MnP (Table 3) and the cyclooctene oxidation reaction yields achieved for the respective solids (Table 6) pointed to a dependence of product yield on the quantity of water in the solid. In other words, water might affect the catalytic behavior of the material in these reactions [103].

In conclusion, the catalytic results presented here suggested that the catalytic properties of the investigated MnP-LDH solids strongly depended on the nature of the LDH support, the M^+2^/M^+3^ molar ratio, and the intercalated anion, which consequently influenced the mode of negatively charged MnP immobilization on the layered crystals surface.

## 4. Conclusions

Layered compounds are a very interesting class of compounds for the immobilization of porphyrins, metalloporphyrins (MP), phthalocyanines, and other macrocyclic complexes. Immobilization may occur on the layered surface or between the layers (intercalation).

Layered double hydroxides (LDHs) have long been investigated as supports for MP immobilization. In recent years, new catalysts have been developed by diverse strategies like LDH exfoliation, LDH restacking, low-polar MP encapsulation into LDH layers intercalated with dodecyl sulfonate, and MP entrapment into LDHs anchored on magnetic particles. The latter alternative facilitates catalyst recovery and reuse because a simple magnet can separate the catalyst from the reaction medium.

Some layered hydroxide salts (LHSs) such as zinc hydroxide nitrate (ZHN) and zinc hydroxide chloride (ZHC) display distinct behavior regarding MP immobilization, including modified selectivity of the supported catalyst during cyclohexane oxidation, which indicates that the support influences the oxidation mechanism. In addition, zinc oxides derived from LHSs exert cooperative effects on immobilized MP during catalytic toluene oxidation.

MPs immobilized on LHSs remain unexplored. Many LHSs can be used for investigative purposes. LHSs may contain Cu^2+^, Co^2+^, Ni^2+^, Mn^2+^, and mixtures of two or even three different divalent metals as well as intercalated hydrophyllic anions (such as NO_3_^−^, Cl^−^, SO_4_^2−^, and CO_3_^2−^) or hydrophobic interlayer anions (like carboxylates, sulfonates, and phosphates). In the case of organic divalent anionic species bearing one negative charge at the end of the ion, grafting of the species to the support is also possible, and pillared derivatives with high surface area may arise. If by only changing the interlayer anion in the support bearing the anionic FeP (e.g., ZHN and ZHC) greatly modifies the oxidation mechanism, more surprises may lie ahead for MP immobilization on LHSs. Moreover, MP immobilization on LHS oxide derivatives can aid the development of novel catalysts for oxidation or photocatalytic reactions.

This work also reported on immobilization of a manganese(III) porphyrin [Mn(TDFSPP)] (MnP) on different LDHs prepared with variable M^+2^/M^+3^ molar ratios (M^+2^ = Mg^+2^ or Zn^+2^ and M^+3^ = Al^+3^) and different intercalated anions (monovalent nitrate and divalent carbonate) to investigate how these variables affected MnP immobilization and catalytic performance. Preliminary catalytic results suggested that composition of the prepared LDHs seemed to impact the catalytic performance of the immobilized MnP. Distinct divalent metals (Mg^+2^ or Zn^+2^) in the LDH elicited different results. The M^2+^/M^3+^ molar ratio in the support correlated positively with the catalytic activity. Probably, changes in the basicity of the support and not the spacing between the positive charges in the layers (*i.e.*, the MP mode of immobilization) influenced the MnP catalytic activity. This proposition is currently under investigation in our research lab.

## Figures and Tables

**Table 1:** Summary of the main MP immobilized in LDH in Section 2.1.

MP	LDH	Use	Reference
Fe or Mn glycol metalloporphyrin	Mg/Al-LDH (nitrate anions)	Oxidation of cyclooctene and cyclohexane	[4]
[Fe(TDFSPP)] and [Fe(TCFSPP)]	Mg/Al-LDH (glycinate anions)	Oxidation of cyclooctene and cyclohexane	[53]
Mn(TPP)OAc	Zn/Al-LDH (dodecyl sulfonate anions)	Epoxidation of alkenes	[54]
CoPcS (phthalocyanine)	Mg/Al-LDH@Fe_3_O_4_ (magnetic)	Oxidation of mercaptans	[55]
[Fe(TDCSPP)]	Mg/Al-LDH (macroporous)	Oxidation of cyclooctene and cyclohexane	[56]
MnTSPP	Mg or Ni/Al-LDH (intercalated porphyrin)	Epoxidation of cyclohexene	[57]
[Fe(TPFPP)Cl]	Mg/Al-LDH (nitrate anions)	Epoxidation of cyclohexene	[58]

**Table 2:** Nomenclature used for the prepared LDH solids with different intercalated anions, basal distance (obtained by XRPD experiments) and calculated “a” parameter.

Mg/Al-Anion	Mg:Al (Molar Ratio)	Basal Distance/(Å)	“a” Parameter/(Å) ^c^	Zn/Al-Anion *	Zn:Al (Molar Ratio)	Basal Distance/(Å)	“a” Parameter/(Ǻ) ^c^
LDH1 ^a^	2:1	7.57	3.036	LDH4 ^a^	2:1	7.38	3.041
LDH2 ^a^	3:1	7.60	3.037	LDH5 ^a^	3:1	7.22	3.052
LDH3 ^a^	4:1	7.67	3.042	*	-	-	-
LDH6 ^b^	2:1	8.66	3.037	LDH9 ^b^	2:1	8.87	3.042
LDH7 ^b^	3:1	8.66	3.038	LDH10 ^b^	3:1	8.87	3.047
LDH8 ^b^	4:1	8.66	3.041	LDH11 ^b^	4:1	8.80	3.054

^a^ Intercalating anion is carbonate; ^b^ Intercalating anion is nitrate and ^c^ Calculation of this parameter is based on the average distance between the metal cations (a = 2d_110_). * It was not possible to synthesize the solid Zn/Al 4:1 with CO_3_^2−^ in a pure phase.

**Table 3:** Percentage (%) of H_2_O loss from LDHs and comparison of the theoretical and calculated M^2+^/M^3+^ ratios.

LDH-CO_3_	%H_2_O	M^2+^/M^3+^ Molar Ratio		LDH-NO_3_	%H_2_O	M^2+^/M^3+^ Molar Ratio	
		Calculated	Theoretical			Calculated	Theoretical
LDH1	19.9	2.3	2	LDH6	7.08	1.8	2
LDH2	7.41	3.5	3	LDH7	3.84	3.3	3
LDH3	9.44	4.6	4	LDH8	6.33	4.4	4
LDH4	3.5	2.2	2	LDH9	4.38	2.0	2
LDH5	8.82	3.5	3	LDH10	2.84	3.4	3
				LDH11	1.16	4.5	4

**Table 4:** Loading values calculated for the solids obtained after immobilization of 100% MnP on the prepared LDHs ^a^.

LDH-CO_3_	Loading ^b^/10^−6^ mol·g^−1^	LDH-NO_3_	Loading ^b^/10^−6^ mol·g^−1^
LDH1 (Mg/Al—2:1)	5.7	LDH6 (Mg/Al—2:1)	4.0
LDH2 (Mg/Al—3:1)	4.6	LDH7 (Mg/Al—3:1)	4.0
LDH3 (Mg/Al—4:1)	6.4	LDH8 (Mg/Al—4:1)	4.1
LDH4 (Zn/Al—2:1)	5.9	LDH9 (Zn/Al—2:1)	4.4
LDH5 (Zn/Al—3:1)	5.3	LDH10 (Zn/Al—3:1)	4.0
		LDH11 (Zn/Al—4:1)	4.1

^a^ MnP immobilization was conducted by dispersing around 700 mg of the LDH solid in 10 mL of solvent and 4 mg of MnP. This suspension was magnetically stirred for 2 h. Then, the resulting solid was centrifuged and washed with methanol and the supernatant was analyzed by UV-Vis spectroscopy in order to quantify the MnP that could have been removed from the matrix by leaching. The loading values were determined indirectly. The light yellow solids MnP-LDH were dried at 60 °C. ^b^ Loading = mol of MnP per 1 gram of solid obtained in the immobilization process.

**Table 5:** Calculated distances between the positive charges of the LDH layers, multiplied by two.

LDHX Solid	d _1,2_ (Å)	d _2,3_ (Å)	d _3,4_ (Å)	d _4,1_ (Å)	d _1,3_ (Å)	d _2,4_ (Å)	As Shown in
LDH1 (Mg/Al–2:1)	10.52	10.52	10.52	10.52	10.52	10.52	Figure 10b
LDH2 (Mg/Al—3:1)	12.15	12.15	12.15	12.15	12.15	21.04	Figure 10c
LDH3 (Mg/Al—4:1)	16.10 *	10.54	16.10 *	10.54	16.10 *	27.88	Figure 10d
LDH4 (Zn/Al—2:1)	10.53	10.53	10.53	10.53	10.53	10.53	Figure 10b
LDH5 (Zn/Al—3:1)	12.21	12.21	12.21	12.21	12.21	21.14	Figure 10c
LDH6 (Mg/Al—2:1)	10.52	10.52	10.52	10.52	10.52	10.52	Figure 10b
LDH7 (Mg/Al—3:1)	12.15	12.15	12.15	12.15	12.15	21.05	Figure 10c
LDH8 (Mg/Al—4:1)	16.09 *	10.53	16.09 *	10.53	16.09 *	27.87	Figure 10d
LDH9 (Zn/Al—2:1)	10.54	10.54	10.54	10.54	10.54	10.54	Figure 10b
LDH10 (Zn/Al—3:1)	12.19	12.19	12.19	12.19	12.19	21.11	Figure 10c
LDH11 (Zn/Al—4:1)	16.16 *	10.58	16.16 *	10.58	16.16 *	27.99	Figure 10d

* Distances coincident with the found value for the distance between the negative charges of MnP.

**Table 6:** Results obtained in the epoxidation reactions cyclooctene.

Catalyst	Run	LDH (M^2+^/M^3+^)	LDH Anion	Epoxide Yield (%) ^a^	Epoxide Yield, Corrected ^d^
MnP ^b^	1			76.3 ± 3.8	62.2
MnP-LDH1	2	2:1	Mg/Al-CO_3_	51.3 ± 3.2	47.3
MnP-LDH2	3	3:1	Mg/Al-CO_3_	71.2 ± 2.9	64.8
MnP-LDH3	4	4:1	Mg/Al-CO_3_	49.9 ± 5.7	46.4
MnP-LDH4	5	2:1	Zn/Al-CO_3_	68.2 ± 4.4	60.3
MnP-LDH5	6	3:1	Zn/Al-CO_3_	77.1 ± 5.4	71.2
MnP-LDH6	7	2:1	Mg/Al-NO_3_	61.2 ± 2.6	55.3
MnP-LDH7	8	3:1	Mg/Al-NO_3_	57.6 ± 3.7	50.4
MnP-LDH8	9	4:1	Mg/Al-NO_3_	50.2 ± 4.1	44.9
MnP-LDH9	10	2:1	Zn/Al-NO_3_	71.7 ± 5.2	62.6
MnP-LDH10	11	3:1	Zn/Al-NO_3_	75.0 ± 3.6	67.6
MnP-LDH11	12	4:1	Zn/Al-NO_3_	73.1 ± 4.1	65.8
LDH1	13	2:1	Mg/Al-CO_3_	4.0 ± 3.8	-
LDH2	14	3:1	Mg/Al-CO_3_	6.4 ± 2.3	-
LDH3	15	4:1	Mg/Al-CO_3_	3.5 ± 4.8	-
LDH4	16	2:1	Zn/Al-CO_3_	7.9 ± 6.3	-
LDH5	17	3:1	Zn/Al-CO_3_	5.9 ± 4.4	-
LDH6	18	2:1	Mg/Al-NO_3_	2.5 ± 5.6	-
LDH7	19	3:1	Mg/Al-NO_3_	7.2 ± 2.2	-
LDH8	20	4:1	Mg/Al-NO_3_	5.3 ± 3.7	-
LDH9	21	2:1	Zn/Al-NO_3_	9.1 ± 5.3	-
LDH10	22	3:1	Zn/Al-NO_3_	7.4 ± 6.1	-
LDH11	23	4:1	Zn/Al-NO_3_	7.3 ± 4.3	-
Control ^c^	24			14.1 ± 1.3	-

^a^ Reaction yield (in percentage) based on the quantity of oxidant (iodosylbenzene) used during the reaction; ^b^ homogeneous catalysis. Epoxide = Cyclooctene oxide; ^c^ Control reaction: reaction performed with iodosylbenzene and cyclooctene only under the same reaction conditions used for run 1; ^d^ Epoxide yield, corrected = (Epoxide yield%–epoxide yield% obtained for the respective LDH without MnP). For the homogeneous catalysis = (%run1 − %run24).

## Supplementary Materials

The following are available online at www.mdpi.com/link/1420-3049/21/3/291/s1, Figure S1: Infrared spectra of the prepared LDHs intercalated with: (**a**) CO_3_^2−^; (**b**) NO_3_^−^.; Figure S2: Thermogravimetric analysis of the synthetized LDHs.
