# Supplementary material for: Recent Advances in Solid Catalysts Obtained by Metalloporphyrins Immobilization on Layered Anionic Exchangers: A Short Review and Some New Catalytic Results
Source: Molecules. 2016 Feb 29;21(3):291. doi: 10.3390/molecules21030291 (PMC6273982; doi:10.3390/molecules21030291)
Supplement: Supplementary file 1 [file molecules-21-00291-s001.pdf]

Supplementary Materials: Recent Advances in Solid Catalysts Obtained by Metalloporphyrins Immobilization on Layered Anionic Exchangers: A Short Review and Some New Catalytic Results

Shirley Nakagaki [1,*], Karen Mary Mantovani [1], Guilherme Sippel Machado [1,2], Kelly Aparecida Dias de Freitas Castro [1,3] and Fernando Wypych [1]

Figure S1 depicts the infrared analyses of the synthesized LDHs.

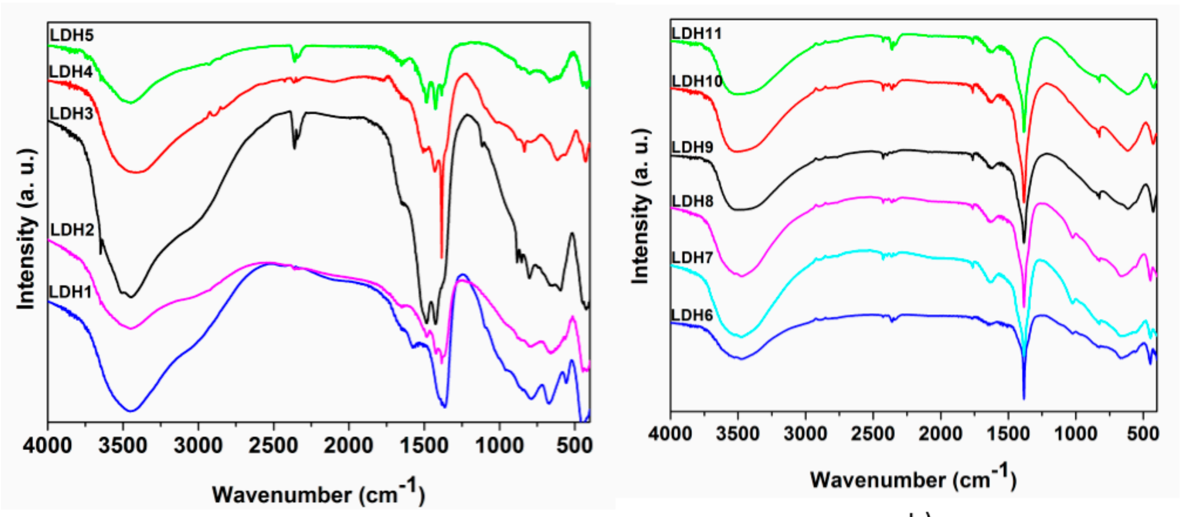

a) b)

Figure S1. Infrared spectra of the prepared LDHs intercalated with: (**a**) CO_3^{2-}; (**b**) NO_3^-.

Figure S2 shows the thermogravimetric analysis of the synthetized LDHs. To estimate the amount of water intercalated and/or adsorbed, the percentage of mass loss in the first heat event for each prepared solid was analyzed. The calculus of the solids chemical composition were made in relation to the percentage of residual oxides mass at the end of analysis.

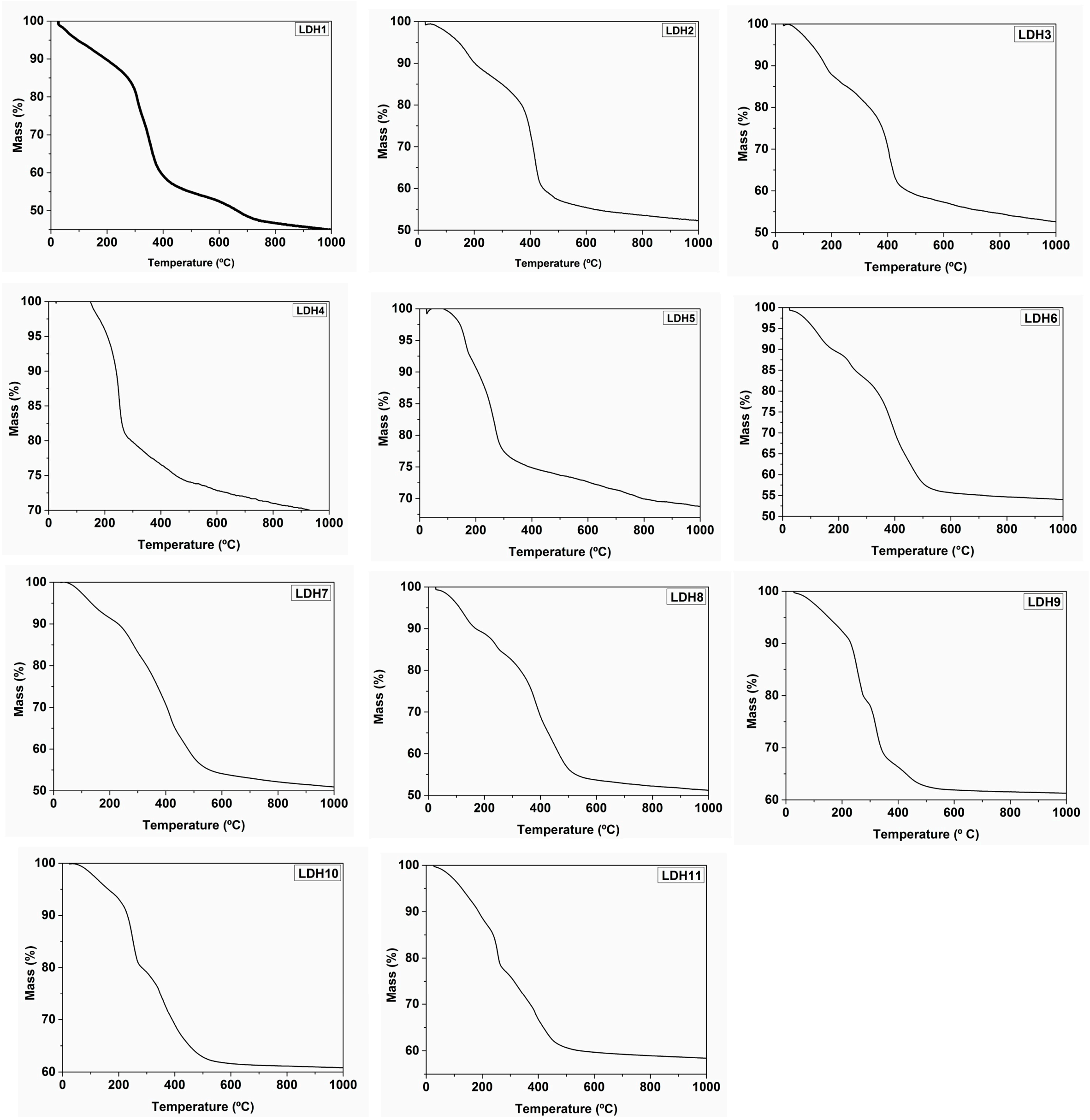

Figure S2. Thermogravimetric analysis of the synthetized LDHs.
